# RNA processing genes characterize RNA splicing and further stratify colorectal cancer

**DOI:** 10.1111/cpr.12861

**Published:** 2020-06-28

**Authors:** Xiaofan Lu, Yujie Zhou, Jialin Meng, Liyun Jiang, Jun Gao, Yu Cheng, Hangyu Yan, Yang Wang, Bing Zhang, Xiaobo Li, Fangrong Yan

**Affiliations:** ^1^ State Key Laboratory of Natural Medicines Research Center of Biostatistics and Computational Pharmacy China Pharmaceutical University Nanjing P.R. China; ^2^ Division of Gastroenterology and Hepatology Key Laboratory of Gastroenterology and Hepatology Ministry of Health Renji Hospital School of Medicine Shanghai Jiao Tong University, Shanghai Institute of Digestive Disease Shanghai P.R. China; ^3^ Department of Urology, The First Affiliated Hospital of Anhui Medical University Institute of Urology & Anhui Province Key Laboratory of Genitourinary Diseases Anhui Medical University Hefei P.R. China; ^4^ Department of Pathology and Urology University of Rochester Medical Center Rochester NY USA; ^5^ Department of Biostatistics The University of Texas MD Anderson Cancer Center Houston TX USA; ^6^ Department of Radiology The Affiliated Nanjing Drum Tower Hospital of Nanjing University Medical School Nanjing P.R. China

**Keywords:** alternative splicing events, colorectal cancer, prognostic value, restricted mean survival, RNA processing genes

## Abstract

**Objectives:**

Due to the limited evaluation of the prognostic value of RNA processing genes (RPGs), which are regulators of alternative splicing events (ASEs) that have been shown to be associated with tumour progression, this study sought to determine whether colorectal cancer (CRC) could be further stratified based on the expression pattern of RPGs.

**Materials and Methods:**

The gene expression profiles of CRCs were collected from TCGA (training set) and three external validation cohorts, representing 1060 cases totally. Cox regression with least absolute shrinkage and selection operator (LASSO) penalty was used to develop an RNA processing gene index (RPGI) risk score. Kaplan‐Meier curves, multivariate Cox regression and restricted mean survival (RMS) analyses were harnessed to evaluate the prognostic value of the RPGI.

**Results:**

A 22‐gene RPGI signature was developed, and its risk score served as a strong independent prognostic factor across all data sets when adjusted for major clinical variables. Moreover, ASEs for certain genes, such as *FGFR1* and the *RAS* oncogene family, were significantly correlated with RPGI. Expression levels of genes involved in splicing‐ and tumour‐associated pathways were significantly correlated with RPGI score. Furthermore, a combination of RPGI with age and tumour stage resulted in significantly improved prognostic accuracy.

**Conclusions:**

Our findings highlighted the prognostic value of RPGs for risk stratification of CRC patients and provide insights into specific ASEs associated with the development of CRC.

## Graphical abstract

Using 774 RNA processing genes, a 22‐gene risk signature was developed and validated. The prognostic value of RNA processing genes was highlighted for risk stratification of colorectal cancer patients and provides insights into specific alternative splicing events associated with the development of colorectal cancer.

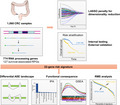

## 1 INTRODUCTION

Colorectal cancer (CRC) is the third most prevalent malignant tumour, accounting for 9% of all cancer‐related fatalities worldwide in men and 8% in women.^1^ Aberrant gene expression profiles play an essential role in the progression of CRC.^2^ A series of processes involved in post‐transcriptional RNA processing can mediate gene expression, including removal of introns through alternative RNA splicing, as well as 5′‐cap and 3′‐end formation.^3^ RNA processing, a determinant factor in translating genotype to phenotype, is pivotal for the DNA damage response, cancer development and chemo‐resistance.^4^, ^5^, ^6^, ^7^ Given that dysregulated expression of RNA processing genes may contribute to abnormalities in RNA expression profiles in CRC patients, systematic examination of the roles that RNA processing factors play in CRC is warranted.

RNA processing factors function in intron removal and regulate alternative splicing events (ASEs) of eukaryotic genes. Aberrant selective RNA processing, especially alternative splicing (AS), facilitates cancer development and progression via alterations in protein structure, nonsense‐mediated mRNA decay, DNA repair defects and genome instability.^8^, ^9^ In CRC, several RNA processing factors, including *HNRNPLL*, *SRSF1*, *SRSF3* and *SRSF6*, have been shown to actively participate in tumour progression and have demonstrated prognostic value, indicating that genetic alterations affecting RNA splicing are associated with CRC pathogenesis.^10^, ^11^, ^12^, ^13^ Recently, Xiong et al analysed seven kinds of ASEs in CRC and linked a selection of ASEs to patient clinical outcomes.^14^ However, to date, the prognostic significance of RNA processing genes serving as regulators of ASEs has not been clearly elucidated.

In this study, we systematically investigated the capability of RNA processing gene expression profiling for the prediction of overall survival in a total of 1060 CRC patients. RNA‐sequencing data from The Cancer Genome Atlas (TCGA) and microarray data from the Gene Expression Omnibus (GEO) database were utilized for the construction and validation of the RNA processing‐related signature. The association between this signature and both AS profiles and clinicopathological variables of CRC patients were further analysed. Eventually, ingenuity pathway analysis (IPA) and gene set enrichment analysis (GSEA) identified that a higher‐risk score in the RNA processing‐related signature was involved in several aspects of tumour progression in CRC patients, including RNA damage and repair, cell death and cell cycle regulation. These results provide novel insights into CRC progression and RNA processing.

## 2 MATERIALS AND METHODS

### 2.1 Study population

Molecular data from patients diagnosed with colorectal cancer were retrieved from TCGA. Transcriptome HTSeq‐count data from the TCGA‐COAD (colon adenocarcinoma) and TCGA‐READ (rectum adenocarcinoma) projects were downloaded from the Genomic Data Commons using R package “*TCGAbiolinks*”,^15^ including 591 fresh‐frozen samples with primary malignancies. Somatic mutation data and patient survival information were downloaded from PanCanAtlas and were filtered for COAD and READ tumour types. Of these TCGA tumour samples, 43 samples whose overall survival (OS) time was less than three months were excluded to enhance the robustness of downstream analyses; corresponding clinicopathological information of the remaining 548 samples was retrieved from cBioPortal (http://www.cbioportal.org/datasets). Another three independent cohorts downloaded from the GEO, including GSE17536,^16^ GSE17538^17^ and GSE38832,^18^ comprising a total of 512 CRCs with known gene expression matrix and corresponding clinicopathological information were utilized to confirm the performance of the prognostic signature. Of these external validation cohorts, gene expression matrices were profiled using the Affymetrix Human Genome U133 Plus 2.0 Array; the same exclusion criteria of OS were followed.

### 2.2 Data pre‐processing for gene expression profiles

For raw data from high‐throughput sequencing, Ensembl IDs for mRNAs were transformed to gene symbols with GENCODE27. The number of fragments per kilobase of non‐overlapped exon per million fragments mapped (FPKM) was computed first and transformed into transcripts per kilobase million (TPM) values, which showed greater similarity to those generating from microarray analysis and were more comparable between samples.^19^ The mRNAs with TPM values less than 1 in over 90% of samples were considered to be noise and removed. For microarray data retrieved from the GEO database, we performed RMA normalization and processing using default settings for background correction and normalization with the R package “*affy*”.^20^ Affymetrix probe ID was annotated to gene symbols according to the GPL570 platform. For multiple probes that mapped to one gene, the mean value of expression was considered.

### 2.3 Collection of RNA processing genes

We collected a total of 929 genes that participated in any procedure engaged in the conversion of at least one primary RNA transcript into at least one mature RNA molecule by searching GO:0006396 term in the AmiGO online database (http://amigo.geneontology.org/amigo). We ultimately collated a total of 774 genes shared in both the TCGA and GEO data sets with sufficiently reliable expression for further analyses.

### 2.4 Identification of the prognostic signature

Univariate Cox regression analysis was performed on the expression matrix of RNA processing genes (RPGs) to first determine genes that were associated with prognosis of CRC patients in the TCGA data set with a relatively loose threshold of *P* < .1. To enhance robustness of the risk signature, the TCGA cohort of 548 samples was randomized into two subsets based on 5‐fold sampling; the training set included 4‐fold CRC samples, and the internal testing set included the rest. Least absolute shrinkage and selection operator (LASSO) penalty was applied to multivariate Cox regression analyses to build an optimal prognostic signature with the minimum number of RPGs. Ten‐fold cross validation was conducted to tune the optimal value of the penalty parameter *λ*, which gives the minimum partial likelihood deviance. Finally, a set of RPGs (*ie* the prognostic signature) and their non‐zero coefficients were identified and used to build an RPG index (RPGI). An RPGI risk score was calculated for each sample via a linear combination of the selected features, weighted by the corresponding coefficients based on the following formula:

$$R_{\text{RPGI}}=\sum_{i=1}^{n}C_{i}\times E_{i}$$

where *C_i_* is the coefficient, *E_i_* is the normalized expression value of each selected gene by log_2_ and *z*‐score transformations, and *R*_RPGI_ represents the risk score for RPGI. Patients were dichotomized into high‐risk (HRisk) and low‐risk (LRisk) groups using the cohort‐specific median RPGI risk score as the cut‐off for each data set.

### 2.5 Bioinformatics analyses

Gene Ontology (GO) and Kyoto Encyclopedia of Genes and Genomes (KEGG) analyses were utilized for gene set annotation, and GSEA was further used to investigate the functional enrichment of risk score‐associated genes using the R package “*clusterProfiler*”.^21^ Differential expression analysis based on TPM values was conducted by the two‐sample Mann‐Whitney U test. The Benjamini‐Hochberg method was used to adjust nominal *P* values (false discovery rate, FDR) for multiple testing. We divided the mean expression of the treatment group (ie HRisk group) by the control group (*ie* LRisk group) to obtain the fold change value. Differentially expressed genes between the two groups were uploaded into IPA software (Qiagen) for core analysis, which described possible disease and bio‐functions enriched in the data set. The biological significance of the IPA was defined as an absolute value of *z*‐score > 2. The presence of infiltrating stromal cells in tumour was estimated with the R package “*ESTIMATE*”.^22^ The population abundance of tissue‐infiltrating immune and stromal cell populations was estimated with the R package “*MCPcounter*” per sample in the TCGA cohort.^23^ The mutation landscape was analysed with the R package “*maftools*” following initial removal of 100 FLAGS genes,^24^, ^25^ and differentially mutated genes were identified by using the mafCompare() function where genes mutated in greater than 5% of CRC samples in the TCGA cohort were considered. Individual consensus molecular subtype (CMS) was predicted with the R package “*CMScaller*” with an FDR threshold of 0.05 by default.^26^ Eight signal transduction pathways related to colorectal carcinogenesis were analysed based on the published literature,^27^ and we referred to a previous report to establish a signature of these eight oncogenic pathways.^28^ We then used the single sample GSEA (ssGSEA) method on these gene sets to generate enrichment scores for each pathway per sample for the TCGA cohort by using the R package “*GSVA*.” Subsequently, we compared the ssGSEA score of each pathway between the two risk groups.

### 2.6 Construction of regulatory network between RNA processing genes and ASEs

We retrieved RNA splicing data from an online archive (http://bioinformatics.mdanderson.org/TCGASpliceSeq). The percent spliced in (PSI) value, which represents the ratio of included transcript reads in the total transcript reads, was used to quantify the ASEs.^29^ To generate as strongly reliable a set of ASEs as possible, we implemented a series of stringent filters (percentage of samples with PSI value ≥ 75 and average of PSI value ≥ 0.05). RPGs with significant changes in expression levels were used to investigate potential association of the differential PSI levels of ASEs between CRCs with lower‐risk (first quartile) and higher‐risk (fourth quartile) scores. In this context, we measured the Pearson correlation coefficient for each RPG–ASE pair; those pairs with absolute correlation coefficients greater than 0.5 and an FDR less than 0.05 were considered significantly correlated. The potential regulatory network was constructed via each significantly correlated pair and visualized via Cytoscape.^30^

### 2.7 Development and verification of a composite Processing‐Clinical prognostic index

Based on the results derived from multivariate analyses, we integrated age (continuous value), tumour stage (divided into early stage [I + II] and advanced stage [III + IV]; binary value), and RPGI risk score to generate a composite Processing‐Clinical prognostic index (PCPI) by applying a Cox proportional hazard regression model to the TCGA cohort; corresponding coefficients derived from the TCGA cohort were then applied to GEO validation sets for further validation. The prognostic value of the PCPI score was compared with that of the RGPI in continuous form according to the concordance index (C‐index) and given by the restricted mean survival (RMS) curve.^31^ The RMS represents the life expectancy at 120 months (10 years) for patients with different risk scores. The performance of risk groups determined by the RGPI risk score was assessed with reference to the RMS time ratio between the HRisk and LRisk groups.^32^ The higher the RMS value, the greater the prognostic difference.

### 2.8 Immunohistochemical analysis

Protein expression data were obtained from the Human Protein Atlas (HPA) (www.proteinatlas.org). These immunohistochemical staining images were used to determine protein expression of the 22 selected genes in both normal and CRC tissues.

### 2.9 Statistical analyses

All statistical analyses were conducted by R3.6.2 using Mann‐Whitney testing for continuous data and Fisher's exact testing for categorical data. Correlation between two continuous variables was measured via Pearson's correlation coefficient. Kaplan‐Meier curves were generated for survival rates of patients, with difference detection based on log‐rank testing. A Cox proportional hazard regression model was used to calculate the hazard ratios (HRs) and 95% confidence intervals (CI) regarding OS. The C‐index was calculated with “*survcomp*” and compared with the “*compareC*” R packages.^33^ The RMS curve and RMS time ratio were estimated with the “*survival*” and “*survRM2*” R packages. For all statistical analyses, a two‐tailed *P* value less than .05 was considered statistically significant.

## 3 RESULTS

### 3.1 Overview of study design

**FIGURE 1:**
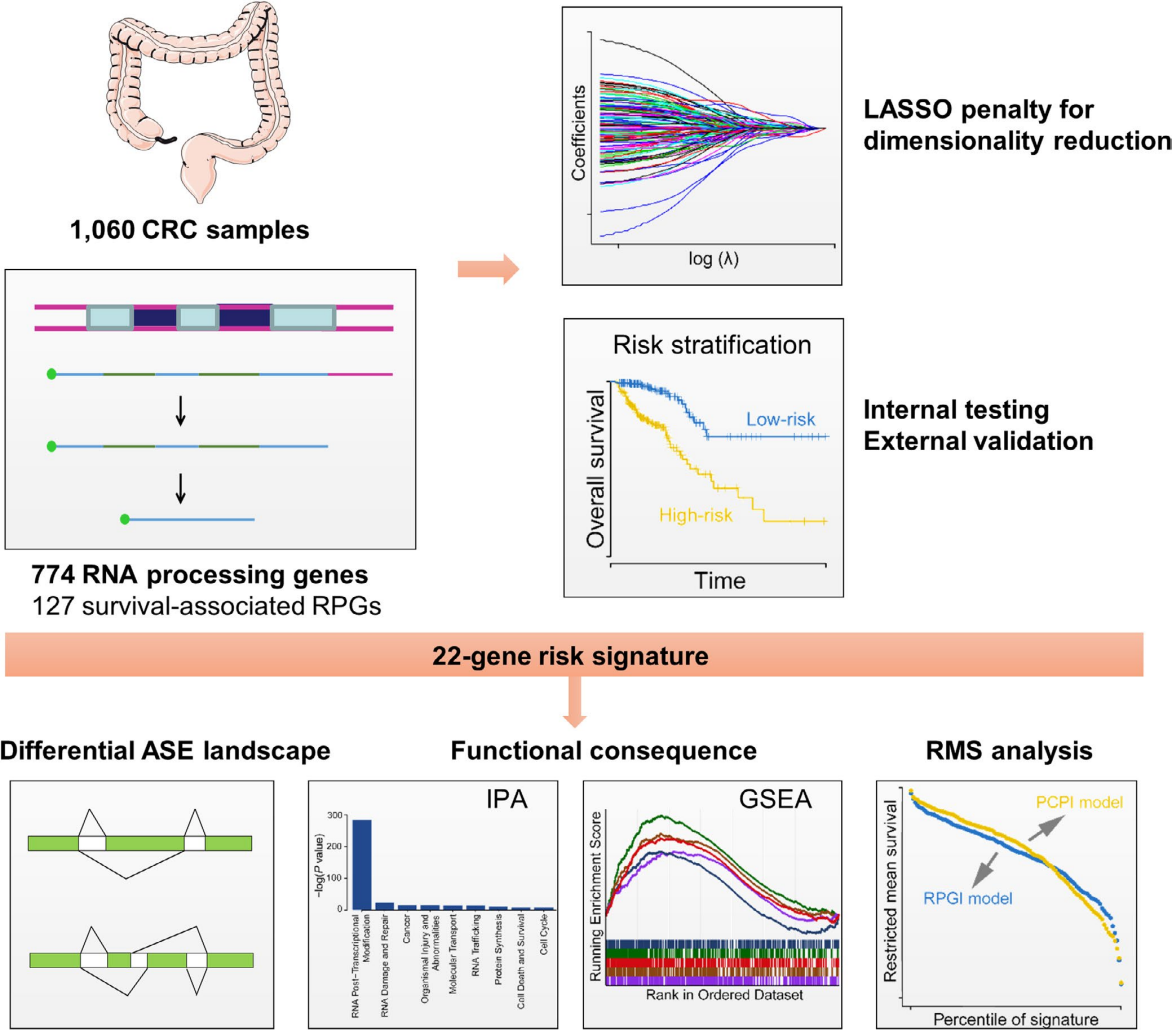
Flow chart of the study design. Using 774 RNA processing genes derived from Gene Ontology (GO: 0006396), we constructed a 22‐gene risk signature in the TCGA cohort that was subsequently validated in three external validation cohorts from GEO. Furthermore, we identified differential splicing events and underlying splicing networks between first and fourth quartiles of risk score. Moreover, pathway annotation by GSEA and IPA provided functional consequences associated with the RNA processing signature. Clinical prognostic value of this signature was highlighted by C‐index and the restricted mean survival (RMS) curve

A total of 1060 patients diagnosed with CRC from four independent data sets were ultimately included in this study; demographic and clinical characteristic descriptions of the different data sets are summarized in Table 1. The entire workflow of this study, including the filtration of RPGs, development and validation of a prognostic signature (*ie* RPGI), the analyses of RPGI‐associated alteration of the ASEs and RNA expression profiles, and the construction of a composite Processing‐Clinical prognostic index (*ie* PCPI), are delineated in Figure 1. A schematic view of RNA processing gene selection and prognostic signature development is depicted in Figure S1.

**TABLE 1:** Demographic and clinic characteristic descriptions for colorectal cancer patients in different data sets

Characteristics^a^	TCGA cohort	Validation set 1	Validation set 2	Validation set 3
Number of samples	548	172	225	115
Median survival time (month) (95% CI)^b^	83.0 (65.7‐NA)	134.9 (65.9‐NA)	134.9 (68.8‐NA)	NA
Number of death (%)	109 (19.9)	69 (40.1)	87 (38.7)	24 (20.9)
Age (years)^c^	66.0 ± 12.5	65.6 ± 13.2	64.6 ± 13.4	—
Gender				
Female	246 (44.9)	79 (45.9)	107 (47.6)	—
Male	302 (55.1)	93 (54.1)	118 (52.4)	—
Tumour stage				
I	91 (16.6)	24 (14.0)	28 (12.4)	18 (15.7)
II	203 (37.0)	57 (33.1)	71 (31.6)	34 (29.6)
III	162 (29.6)	56 (32.6)	75 (33.3)	38 (33.0)
IV	73 (13.3)	35 (20.3)	51 (22.7)	25 (21.7)
CMS (Predicted)				
CMS1	85 (15.5)	29 (16.9)	37 (16.4)	21 (18.3)
CMS2	154 (28.1)	48 (27.9)	44 (19.6)	29 (25.2)
CMS3	84 (15.3)	24 (14.0)	32 (14.2)	19 (16.5)
CMS4	160 (29.2)	49 (28.5)	65 (28.9)	33 (28.7)

^a^ Sum of frequency numbers may not equal to the total sample size due to missing or unpredictable values.

^b^ Median survival time is incalculable because the mortality at the last follow‐up time is less than 50%.

^c^ Age is represented as mean ± standard deviation.

### 3.2 Prognostic value of RPGs and their biological function in CRCs

**FIGURE 2:**
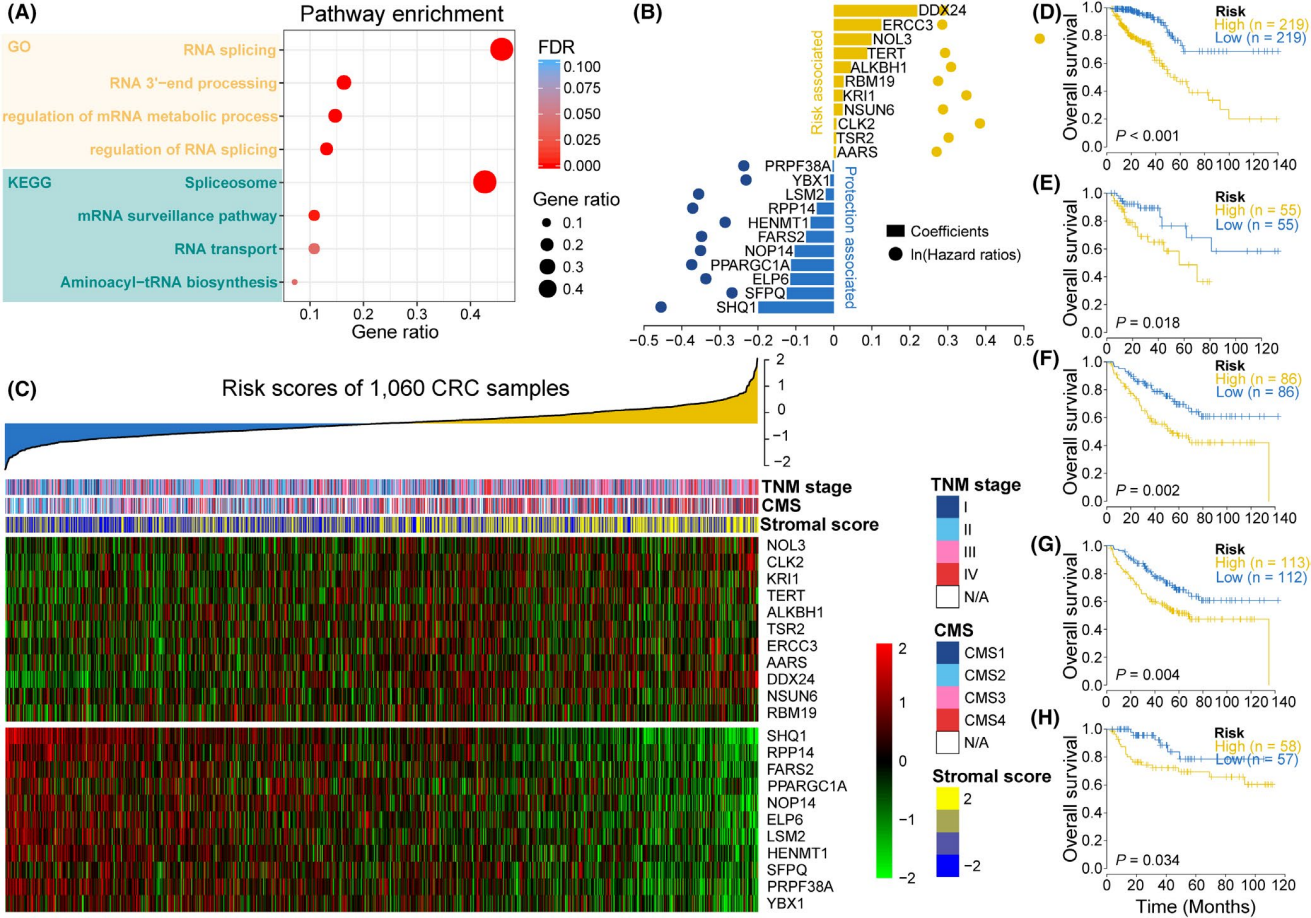
Prognosis‐associated RPG expression profiles in CRCs. A, Dot‐plot showing the pathway enrichment of 127 overall survival‐related RPGs by GO and KEGG analyses. B, Multivariate Cox regression analysis with LASSO penalty identified 22 prognosis‐associated RPGs, which were used to construct an RNA processing gene index (RPGI). Yellow items indicate risk‐associated genes; blue items indicate protection‐associated genes. Corresponding coefficients from multivariate Cox regression using LASSO and HRs are depicted by horizontal bars and dots, respectively. C, Heatmap showing the expression patterns of 22 prognosis‐associated RPGs for the entire 1060 CRC sample set sorted by RPGI risk score in ascending order. Top panel, risk‐associated genes; bottom panel, protection‐associated genes. Individual stromal score, predicted CMS, TNM stage and RPGI risk score are also annotated above the heatmap. Kaplan‐Meier overall survival curves with difference detection of log‐rank testing for patients from the TCGA training set, TCGA internal testing set, and three external validation sets are portrayed in (D)‐(H), respectively. Patients were divided into different risk groups based on a cohort‐specific median cut‐off value of RPGI risk score

We evaluated the prognostic effect of the 774 RPGs and identified 127 genes that were associated with CRC patient OS (Table S1). Among these, 70 RPGs were risk‐associated because the corresponding HRs were greater than 1, while the remaining 57 genes were considered protection‐associated. Since these RPGs represent a grouping of genes that participate in any step involved with the conversion of at least one primary RNA transcript into at least one mature RNA molecule, we used GO analysis to identify the more explicit biological processes that these prognosis‐related RPGs are enriched in. We found that they were relevant to such key biological functions as RNA splicing, RNA 3’‐end processing, regulation of RNA splicing and regulation of mRNA metabolic process, among others (all FDR < 0.001; Figure 2A). We further used KEGG analysis for annotation, and the results indicated that pathways involved in the spliceosome, mRNA surveillance pathway, RNA transport and aminoacyl‐tRNA biosynthesis were closely associated with these RPGs (all FDR < 0.05; Figure 2A).

### 3.3 Feature selection and prognostic signature building

To readily and efficiently categorize clinical outcomes of CRC patients via RPGs, we applied a LASSO penalty with multivariate Cox regression analysis to the TCGA training set and identified 22 features with non‐zero coefficients (Figure 2B, Figure S2A,B). These LASSO‐selected features were used to build a prognostic signature, the RPG index (RPGI), and corresponding RPGI risk scores were computed for all data sets. All 1060 CRC samples were further dichotomized into high‐risk (HRisk) and low‐risk (LRisk) groups, and each sample was predicted to be one of the four CMS (Figure S3A‐D). Interestingly, the HRisk group of the TCGA cohort had more tumour protein 53 (*TP53*) mutations (*P* = .024), and CMS4 especially (*P* < .001; Figure S4A). We then pooled all 1060 CRCs samples together and found that almost all 22 LASSO‐selected features were significantly differentially expressed between the two risk groups (Figure S4B‐C). Moreover, advanced tumour stage (ie stage III and stage IV) was enriched in the HRisk group (57.4% vs 41.5%, *P* < .001; Figure 2C, Figure S5A). We further verified that CMS4, featuring stromal activation,^34^ was dramatically enriched in the HRisk group (46.8% vs 20.3%, *P* < .001; Figure 2C, Figure S5B), which was consistent with the higher enrichment identified in a previously reported stromal score^22^ (*P* < .001; Figure S5C).

In all data sets, we found that the LRisk group had a significantly more favourable prognosis than the HRisk regarding OS (TCGA training set: *P* < .001, HR = 0.22, 95% CI: 0.13‐0.38; TCGA testing set: *P* = .018, HR = 0.38, 95% CI: 0.16‐0.88; validation set 1: *P* = .002, HR = 0.47, 95% CI: 0.29‐0.77; validation set 2: *P* = .004, HR = 0.53, 95% CI: 0.35‐0.82; validation set 3: *P* = .034, HR = 0.38, 95% CI: 0.15‐0.96; Figure 2D‐h). RMS time ratios ranging from 0.62 to 0.84 were observed in the four data sets (TCGA: *P* < .001; Validation set 1: *P* = .008; Validation set 2: *P* = .015; Validation set 3: *P* = .075; Table 2). To further investigate the prognostic performance of the RPGI risk score with adjustment for major clinical variables, including tumour stage and patient age (an exception for validation 3 due to a lack of age records), we performed multivariate Cox regression analysis and found that RPGI risk score was a significant independent prognostic factor for CRC patients (Table 3).

**TABLE 2:** Restricted mean survival (RMS) time ratio between two risk groups in different data sets

Data set	*N*_HRisk_	*N*_LRisk_	RMS_HRisk_ (95% CI)^a^	RMS_LRisk _(95% CI)^a^	RMS ratio (95% CI)^b^	*P*
TCGA cohort	274	274	66.60 (55.07‐78.12)	107.22 (95.86‐118.58)	0.62 (0.51‐0.76)	<.001
Validation set 1	86	86	73.58 (61.08‐86.09)	97.81 (86.90‐109.72)	0.75 (0.61‐0.93)	.008
Validation set 2	113	112	78.02 (66.98‐89.07)	97.31 (86.74‐107.88)	0.80 (0.67‐0.96)	.015
Validation set 3	58	57	76.27 (65.01‐87.53)	90.71 (79.79‐101.63)	0.84 (0.70‐1.02)	.075

^a^ RMS time: months.

^b^ RMS ratio = RMS_HRisk_/RMS_LRisk_.

**TABLE 3:** Multivariate Cox proportional hazard regression in TCGA cohort and three GEO validation data sets

Data set	RPGI risk score		Tumour stage		Age	
	HR (95% CI)	*P*	HR (95% CI)	*P*	HR (95% CI)	*P*
TCGA cohort	4.21 (2.74‐6.47)	<.001	2.93 (1.89‐4.52)	<.001	1.03 (1.02‐1.05)	<.001
Validation set 1	1.84 (1.20‐2.81)	.004	4.10 (2.29‐7.34)	<.001	1.02 (1.00‐1.04)	.051
Validation set 2	2.06 (1.30‐3.24)	.002	3.88 (2.29‐6.57)	<.001	1.02 (1.00‐1.04)	.028
Validation set 3	3.28 (1.41‐7.60)	.006	NA^a^	NA^a^	—^b^	—^b^

^a^ All patients with advanced tumour stage (III + IV) died at the end of follow‐up.

^b^ No record.

We further performed immunohistochemical analysis of the 22 identified genes in The Human Protein Atlas, and we found that the protein products of the risk‐associated genes showed higher expression levels in CRC samples compared with adjacent normal tissues (Figure S6A). In contrast, protein expression of the protection‐associated genes showed the opposite trend (Figure S6B). These results may support the functional relevance of the identified 22 RPGs in CRC patients.

### 3.4 Correlation of RPGI risk score with immunity and oncogenic pathways

We used the MCPcounter algorithm to compare tumour immune microenvironments (TIMEs) between the HRisk group and the LRisk group (Figure S7A). We found significant elevations in the proportion of endothelial cells and fibroblasts in the HRisk group (both *P* < .01), whereas the proportions of CD8 + T cells and NK cells were comparable between the two groups (Figure S7B). We then estimated the enrichment score of eight oncogenic pathways (Figure S7C). We found that *HIPPO*, *NOTCH*, *TGF‐β*, *RTK/RAS* and *Wnt* pathways were significantly enriched in the HRisk group (all *P* < .01), while the *TP53* pathway was significantly downregulated in the HRisk group (*P* < .01), although this might be due to the frequent mutations in TP53 in this group. These results indicated an activation of stromal components in TIME of high‐risk patients together with activated oncogenic pathways based on the proposed signatures, which likely contributed at least partially to the poorer prognosis in these patients.

### 3.5 Functional enrichment of genes that were associated with RPGI risk score

**FIGURE 3:**
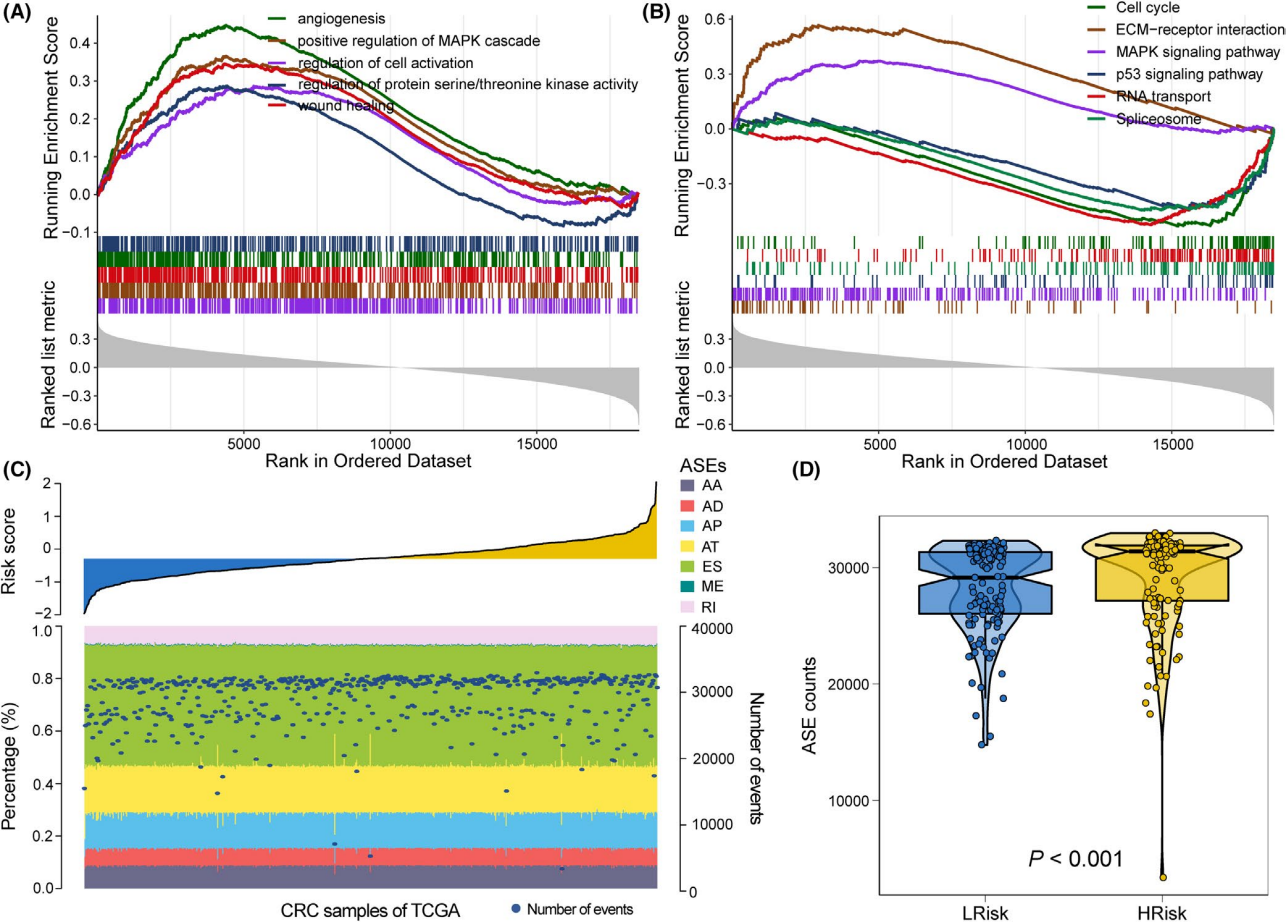
Risk score‐related functional pathways and alternative splicing profile analysis in CRCs with lower‐ (first quartile) or higher‐risk (fourth quartile) scores. A, GSEA of GO for risk scores based on pre‐ranked Pearson's correlation coefficients of risk score‐associated mRNAs. B, GESA of KEGG analysis for risk scores based on pre‐ranked Pearson's correlation coefficients of risk score‐associated mRNAs. C, Proportions of alternative spliced events (ASEs) in 548 TCGA CRC samples sorted by increased risk score. Bars indicate the proportion of each ASE type. Dark blue dots indicate the number of ASEs in each sample. The risk scores in ascending order are shown at the top panel. D, The absolute numbers of all ASEs were compared in CRCs with lower‐ or higher‐risk (both, n = 137) scores

Given that RPGs are the primary elements manipulating the life cycle of RNAs in eukaryotes, we subsequently assessed how the RPGI could mediate RNA expression profiles. In this context, we correlated the RPGI risk score with all robustly expressed mRNAs and generated a pre‐ranked list sorted by Pearson correlation coefficient. We then performed GSEA and found that RPGI risk score was closely associated with dysregulation of the cell cycle, wound healing, angiogenesis and protein serine/threonine kinase activity based on GO terms (all FDR < 0.01; Figure 3A). GSEA of KEGG also revealed dysregulation of the extracellular matrix (ECM)‐receptor interaction, *MAPK* and *p53* signalling pathways, spliceosomes and RNA transport (all FDR < 0.01; Figure 3B). IPA indicated that several biological functions were significantly associated with RPGI risk score, including RNA damage and repair, cell cycle, cell death and RNA trafficking (all *P* < .001; Table 4).

**TABLE 4:** Top enriched diseases and bio‐functions (IPA) associated with risk signature

Disease/bio‐function^a^	*P*	Number of molecules
RNA post‐transcriptional modification	3.47 × 10^−286^	257
RNA damage and repair	1.17 × 10^−24^	55
Cancer	3.66 × 10^−16^	414
Organismal injury and abnormalities	2.19 × 10^−16^	421
Molecular transport	3.92 × 10^−15^	36
RNA trafficking	3.92 × 10^−15^	25
Protein synthesis	6.49 × 10^−12^	53
Cell death and survival	1.16 × 10^−9^	75
Cell cycle	1.23 × 10^−9^	65
Cellular growth and proliferation	4.24 × 10^−7^	48

^a^ Differentially expressed genes between high‐ and low‐risk groups, as determined by RNA‐Seq, were uploaded into Ingenuity Pathway Analysis software to determine the most enriched biological functions underlying the risk signature.

### 3.6 Association between RPGI risk score and ASEs

RNA splicing activities are dominated by RPGs, and we have demonstrated that prognosis‐associated RPGs are closely correlated with RNA splicing‐related activities. Therefore, we comprehensively characterized ASEs in CRCs with different RPGI risk scores. A large number of ASEs within seven categories, including alternate acceptor site (AA), alternate donor site (AD), alternate promoter (AP), alternate terminator (AT), exon skip (ES), mutually exclusive exons (ME) and retained intron (RI), were identified per CRC sample; the proportion of these ASE categories in CRCs varied dramatically, from 0.3% to 45.8% (Figure 3C). Although the proportional pattern of each ASE type was similarly shared for all CRCs, the amount of each of the ASEs showed significant positive correlation with the RPGI (ρ = 0.22, *P* < .001 by Spearman's correlation analysis; Figure 3C), and the amount of detected ASEs was significantly higher in CRCs with a higher‐risk (fourth quartile, n = 137) score compared to those with a lower‐risk (fourth quartile, n = 137) score (*P* < .001, Figure 3D).

**FIGURE 4:**
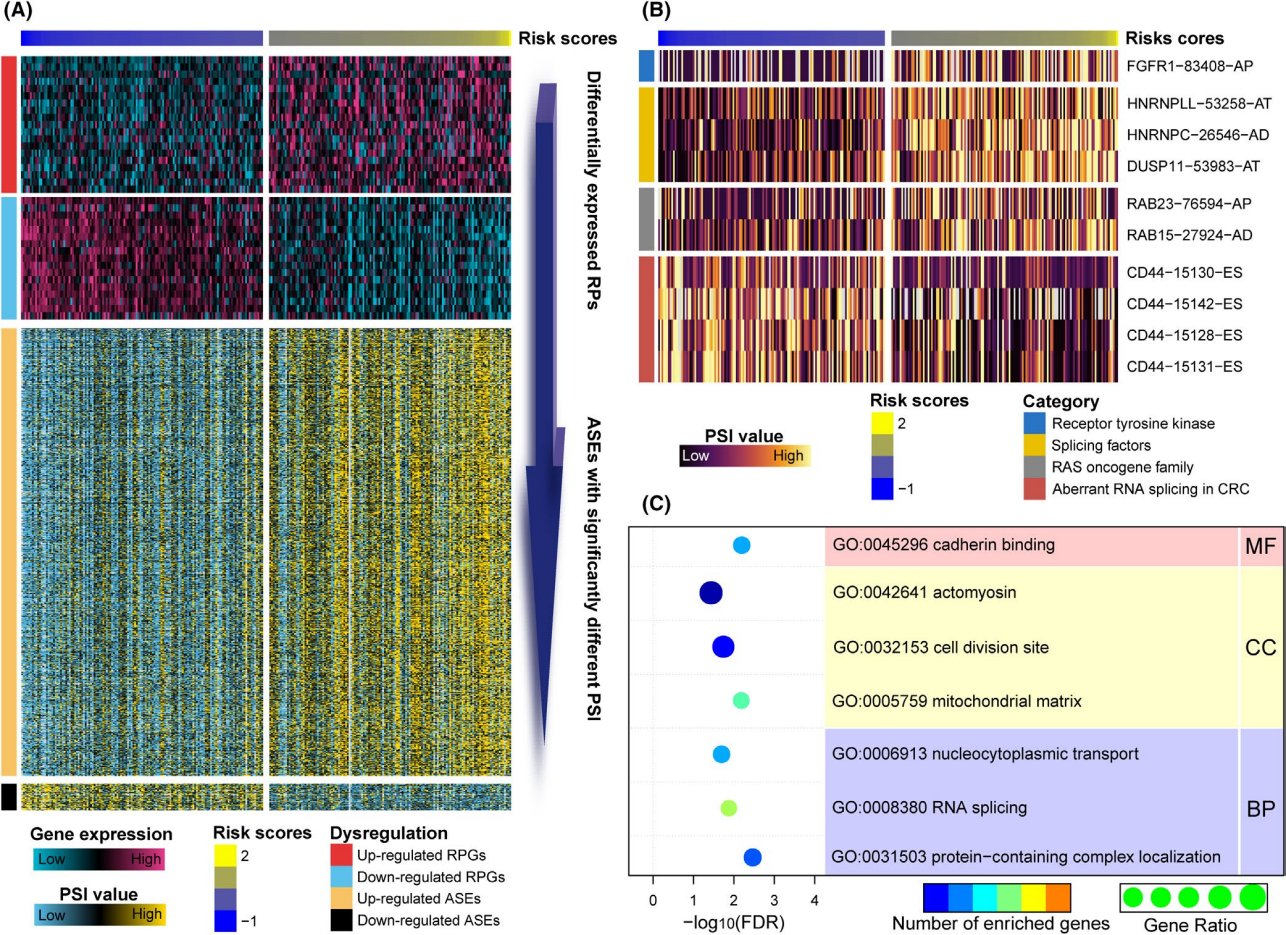
Differential RPGs and ASEs in CRCs with lower‐ or higher‐risk scores. A, Heatmaps displaying the expression levels of RPGs (top panel, peach and blue colour scale) and PSI value of ASEs with significant differences between lower‐ and higher‐risk scores (bottom panel, yellow and blue colour scale). B, Representative ASEs with differential PSI values between CRC lower‐ and higher‐risk groups. C, GO functional annotation of spliced genes with differential PSI values between the lower‐ and higher‐risk CRC groups

We further identified differentially expressed RPGs (absolute fold change > 1.5 and FDR < 0.05; Table S2) and ASEs with significantly different PSI levels (absolute fold change > 1.5 and FDR < 0.05; Table S3) in CRCs with lower and higher RPGIs. In total, 701 ASEs for 623 genes with increased PSI in higher‐risk CRCs were identified, compared to only 42 ASEs for 39 genes with decreased PSI (Figure 4A). We found that genes involved in the RAS oncogene family (*eg RAB15* and *RAB23*), various splicing factors (*eg DUSP11*, *HNRNPLL*, *HNRNPC*), aberrant RNA splicing in CRC (*eg CD44*) and receptor tyrosine kinase signalling (*eg FGFR1*) were differentially spliced among CRCs with lower and higher RPGIs (Figure 4B). To further examine the role(s) of alternative splicing in CRCs, we performed GO analysis for all differential spliced genes in CRCs with lower‐ and higher‐risk scores. Generally, genes that had differential PSI levels were principally related to protein‐containing complex localization, RNA splicing, nucleocytoplasmic transport for biological process, mitochondrial matrix, cell division site, actomyosin for cellular component and cadherin binding for molecular function (all FDR < 0.05; Figure 4C). For these ASEs with markedly different PSIs, we found that the frequency of all ASE types (except for ME, which has the lowest proportion) was significantly altered (*P* < .001 for AP, AT, AD and ES; *P* < .05 for AA and RI; Figure S8) compared to background ASEs, which suggested that the presence of altered ASEs is relevant for the prognosis of CRC patients.

**FIGURE 5:**
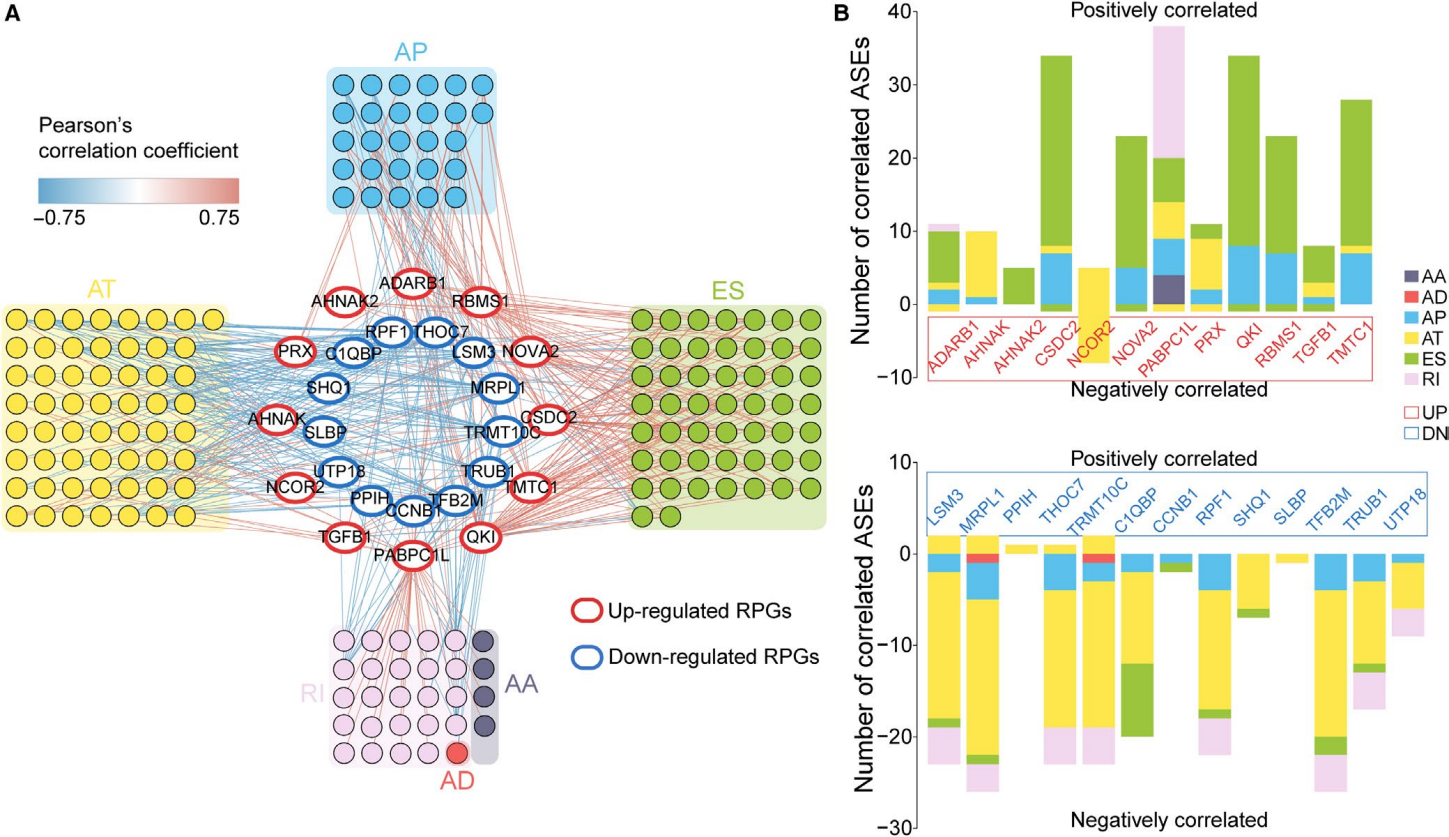
ASE networks and RPGs. A, Labelled circles in the centre represent differentially expressed RPGs. Red ellipses indicate upregulated RPGs in CRCs with higher‐risk scores, whereas blue ellipses indicate downregulated RPGs. Coloured circles connected to RPGs by red or blue lines represent distinct types of differential ASEs. The red connecting lines represent positive correlations, while blue connecting lines represent negative correlations. B, Numbers of ASEs significantly correlated with upregulated (top panel) or downregulated (bottom panel) RPGs. ASE type ME is absent due to its failure to pass the correlation threshold

Subsequently, we examined potential regulatory networks involved among the significantly altered 36 RPGs and 743 ASEs, and constructed a network with 453 pairwise correlations that ultimately involved 25 differential RPGs and 164 associated differential ASEs (Figure 5A, Table S4). The 25 RPGs regulated different numbers of ASEs, which ranged from 5 to 39 for 12 overexpressed RPGs compared to 1 to 28 for 13 under‐expressed RPGs. For RPGs with increased expression, we found that *PABPC1L* regulated a substantial number of ASEs, especially for RI, and *CSDC2* and *QKI* were highly correlated with ES; in contrast, *AHNAK2* regulated fewer ASEs. Among RPGs with decreased expression in CRCs, *LSM3*, *MRPL1*, *THOC7*, *TRMT10C*, *C1QBP*, *RPF1* and *TFB2M* regulated a markedly greater number of ASEs—especially for AT—whereas only a few ASEs were regulated by *PPIH* and *SLBP* (Figure 5B).

### 3.7 Combining RPGI with clinical characteristics

**FIGURE 6:**
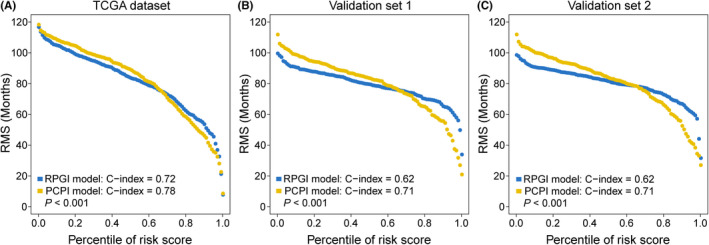
RMS curves for RPGI and the integrated PCPI scores are plotted for: A, the TCGA cohort, B, validation set 1 and C, validation set 2. Each point represents the RMS time of corresponding RPGI and PCPI scores. The RMS curves show a larger slope in all three data sets for PCPI, indicating superior estimation of survival with PCPI. C‐indexes for RPGI and PCPI are also provided. *P* values represent the difference between the two models in terms of C‐index

In addition to RPGI risk score, we also affirmed that clinical characteristics (*ie* age and tumour stage) served as independent prognostic factors, which could have complementary values (Table 3). To further improve the prognostic accuracy, we combined RPGI risk scores with these major clinical variables using the coefficients generated from multivariate Cox regression analysis in the TCGA cohort and derived a PCPI as follows: PCPI = 1.44 × RPGI + 1.07 × stage + 0.03 × age; such an integrated model of PCPI was further applied to the TCGA cohort and validated in validation sets 1 and 2 where full clinical information was available. Significant improvement in estimation of survival was achieved with the continuous form of PCPI relative to RPGI (C‐index: 0.78 vs 0.72 in the TCGA cohort, *P* < .001; C‐index: 0.71 vs 0.62 in validation set 1, *P* < .001; C‐index: 0.71 vs 0.62 in validation set 2, *P* < .001; Figure 6A‐C).

## 4 DISCUSSION

In this study, we linked genomic expression patterns of RPGs with patient clinical outcomes, the alternative mRNA splicing landscape, molecular characteristics and pathway enrichment in CRC. We further constructed a prognostic signature for risk stratification and identified underlying biological functions associated with higher‐risk scores via IPA. The identified altered RPG expression pattern, in combination with various clinical parameters, reliably demonstrated accurate prognostic predictions for CRC patients.

Increasing numbers of anecdotes have suggested that RNA processing, the molecular events by which primary transcripts become mature RNA, plays a critical role in CRC carcinogenesis.^4^, ^13^, ^35^ The roles several famous splicing variants play in tumour progression, such as *CD44*, have been well studied.^36^, ^37^ In this study, we observed that *CD44* was differentially spliced in the lower‐ and higher‐risk groups. Additionally, several genes involved in mRNA splicing, the *RAS* oncogene family and receptor tyrosine kinase signalling also showed significant differential PSIs between the first and fourth quartiles of the risk score. Interestingly, several splicing factors, such as *DUSP11*, *HNRNPLL* and *HNRNPC*, were differentially spliced as a result of aberrant RPG expression profiles, as revealed by high RPG signature scores. Greater numbers of ASEs were identified in the high‐risk group, which was consistent with a previous pan‐cancer report that greater numbers of ASEs were detected in tumour samples compared to normal samples.^9^

Misregulated RNA expression profiles, including autophagy‐related gene sets,^38^ metabolism‐associated gene sets,^39^ immune gene sets,^40^ hypoxia‐associated gene sets,^41^ microRNAs^42^ and long non‐coding RNAs,^43^ have all been shown to affect disease progression and prognosis in CRC. In this study, we demonstrated that dysregulation of RPGs could allow the stratification of CRC patients based on different outcomes. Moreover, we found several splicing‐ and tumour‐associated pathways were enriched with increased risk scores, such as RNA damage and repair, cell cycle regulation, angiogenesis, spliceosome, *p53* and *MAPK* signalling pathways. In reality, the *MAPK* signalling pathway and RNA splicing are inextricably linked with each other. More specifically, the *Ras/MAPK* pathway was regulated by alternative splicing with regard to variants of *EGFR*, *BRAF*, *p19‐* or *p21‐Ras*, *MEK1b* and *ERK1c* ^44^; activation of the *MAPK* pathway also required serine/arginine‐rich splicing factor 1 (*SRSF1*), a splicing factor that can promote tumorigenesis in CRC.^45^, ^46^

Among the 22 survival‐related RPGs in the risk signature, several have previously been reported to have substantial effects on tumorigenesis. The apoptosis repressor with caspase recruitment domain (*ARC*, also termed *NOL3*) can be induced by hypoxia and further promote carcinogenesis by reducing apoptosis in CRC cell lines.^47^ Human telomerase reverse transcriptase (*TERT*) expression has demonstrated prognostic value for predicting recurrence in papillary thyroid carcinomas,^48^ and *TERT* has been found to be overexpressed in the right colon compared to the left,^49^ indicating worse prognosis in CRC. Both papillary thyroid carcinoma and right colon cancer are characterized by *BRAF* V600E mutation, which might interact with TERT expression and TERT mutation for the promotion of tumour invasion.^50^, ^51^ The elongator complex protein 6 (*ELP6*) gene encodes an elongator subunit, which is reportedly capable of controlling cell migration and melanoma tumorigenesis.^52^ PTB‐associated splicing factor (*PSF*), also known as splicing factor proline‐ and glutamine‐rich (*SFPQ*), is a PPARγ‐interacting protein involved in RNA processing and DNA repair process,^53^ and serves as a regulator of apoptosis in colon cancer cell lines.^54^ These results indicate that our study protocol may be able to identify novel carcinogenesis‐associated RPGs; future studies of these prognostic RPGs may identify novel mechanisms underlying RNA processing and overall CRC progression.

Several strengths of this study warrant specific focus. First, we analysed a large sample size of 1060 CRC patients with either RNA‐Seq or microarray data, which indicates that our analysis conclusions are likely highly reliable, robust and independent of specific expression quantitative platform. This also suggests the possibility of future verification of the risk signature in additional cohorts. Second, we used restricted mean survival time, an alternative summary measure of survival time distributions that does not rely on the proportional hazards assumption to demonstrate the clinical utility of the RPG risk signature, which is robust and more clinically interpretable.^55^ However, as with all investigations, certain limitations are present as well. Both validation cohorts were derived from microarray platforms, and additional validation in a prospective cohort using an RNA‐Seq platform is warranted. Additionally, further experimental results regarding these prognosis‐related RPGs are required to elucidate the mechanisms underlying RNA processing and CRC tumorigenesis.

In summary, we identified the prognostic value of specific genes associated with RNA processing in CRC and propose a 22‐RPG signature for risk classification of CRC patients. We further identified differential ASEs, bio‐functions, signalling pathways, and clinical features underlying the RPG risk signature. Combining data concerning age, tumour stage and risk signature could further improve prognosis prediction in CRC patient samples.

## CONFLICT OF INTEREST

The authors have no conflict of interest.

## ETHICAL STATEMENT

As the data (TCGA and GEO data sets) are publicly available, no ethical approval is required.

## AUTHOR‘S CONTRIBUTION

Conceptualization, X‐FL and FY; Methodology, X‐FL, YZ and JM; Software, X‐FL and LJ; Formal analysis, X‐F.L, YZ, JM, and LJ; Investigation, X‐FL, YC, YW, BZ, and HY; Writing–original draft, X‐FL and JG; Visualization, X‐FL and YZ; Funding acquisition, X‐BL and FY; Supervision, X‐BL and FY

## Supporting information

Fig S1

Fig S2

Fig S3

Fig S4

Fig S5

Fig S6

Fig S7

Fig S8

Table S1‐S4

## Data Availability

Raw data for this study were generated at TCGA with cancer type of COAD and READ, and GEO database with Series ID of GSE17536, GSE17538, and GSE38832. Derived data supporting the findings are available from the corresponding authors [XL] or [FY] on reasonable request.
