# Supplementary figures and images for: RNA processing genes characterize RNA splicing and further stratify colorectal cancer
Source: Cell Prolif. 2020 Jun 28;53(8):e12861. doi: 10.1111/cpr.12861 (PMC7445406; doi:10.1111/cpr.12861)

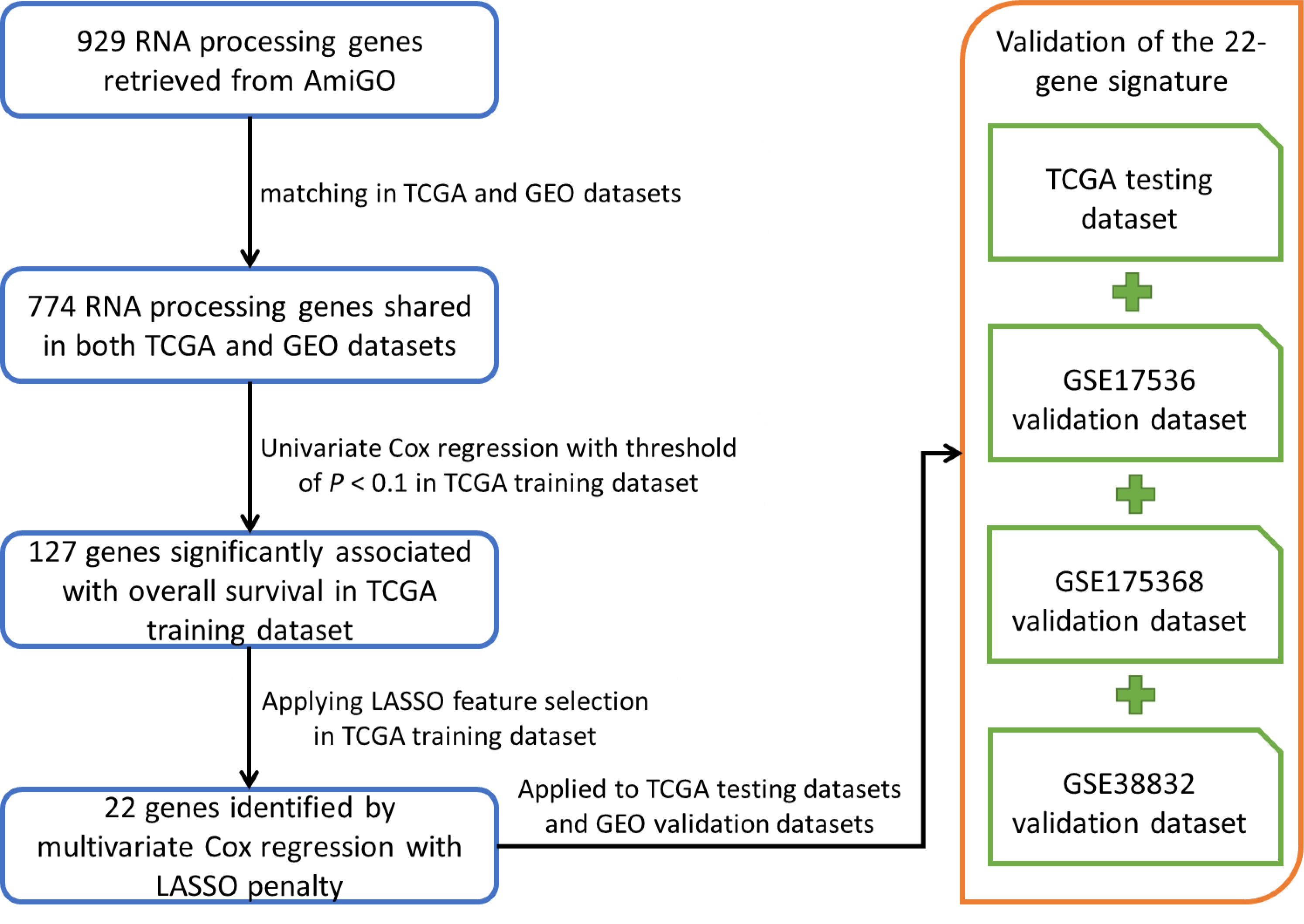

Supplement: Supplementary file 1 — Fig S1 [file CPR-53-e12861-s001.tif]

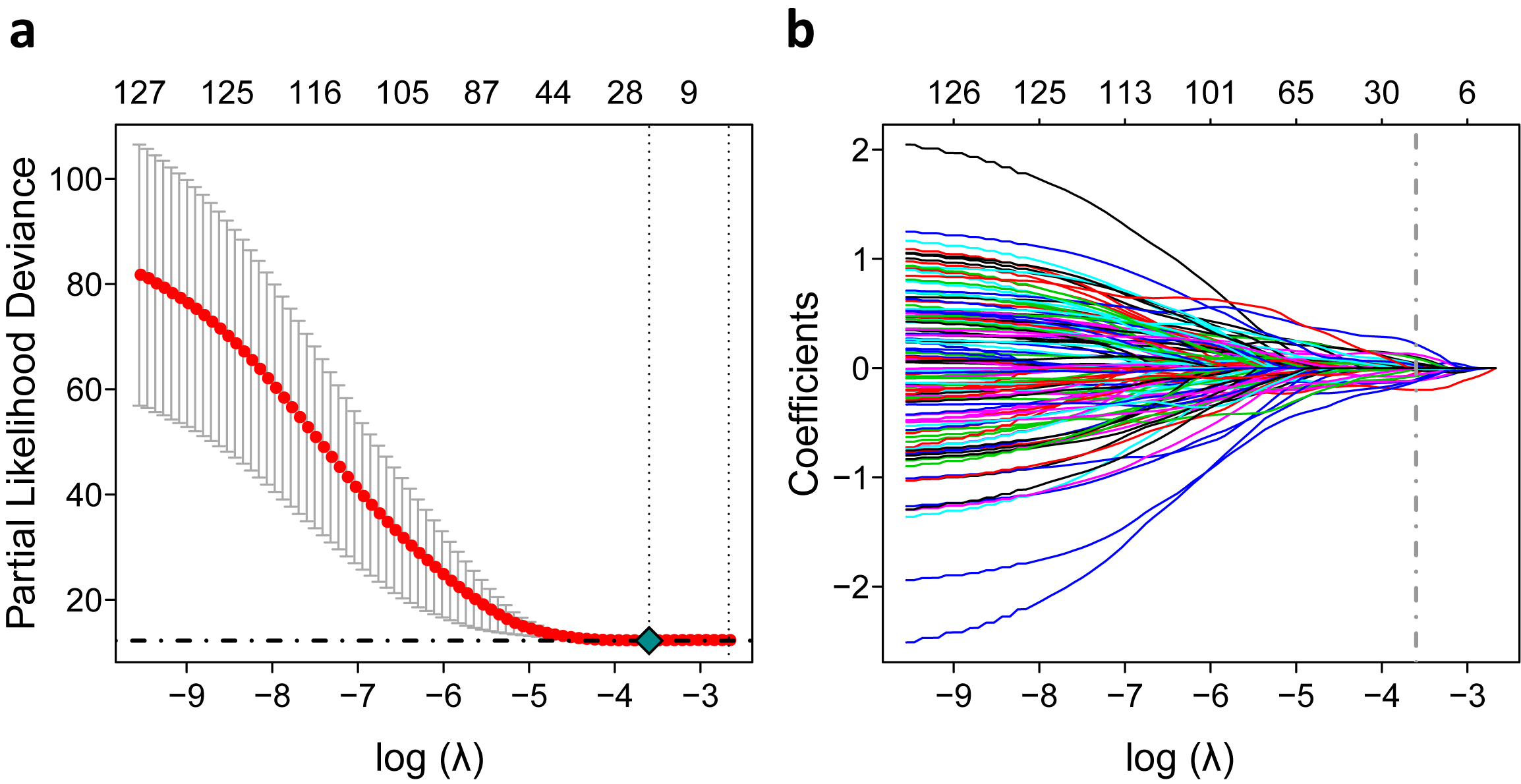

Supplement: Supplementary file 2 — Fig S2 [file CPR-53-e12861-s002.tif]

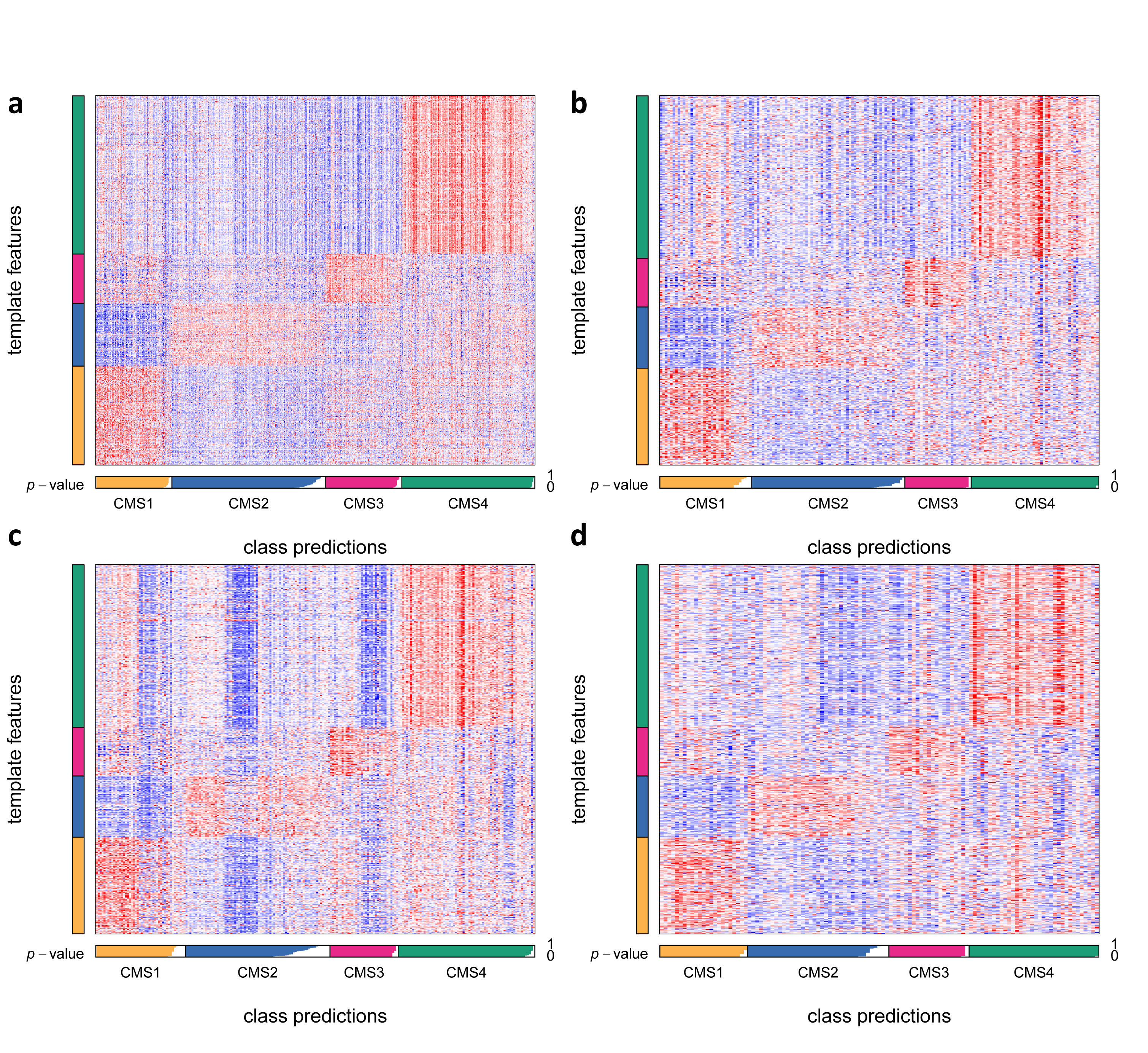

Supplement: Supplementary file 3 — Fig S3 [file CPR-53-e12861-s003.tif]

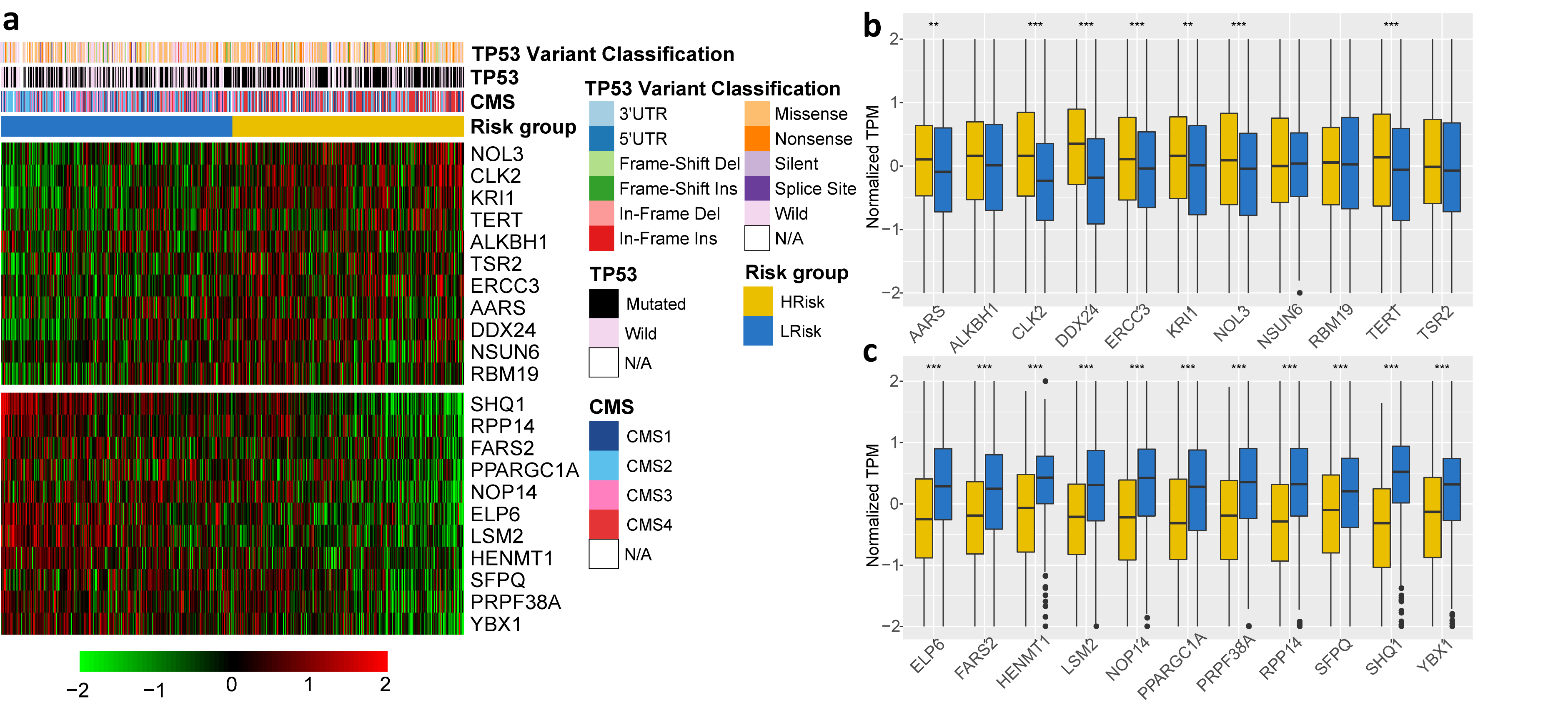

Supplement: Supplementary file 4 — Fig S4 [file CPR-53-e12861-s004.tif]

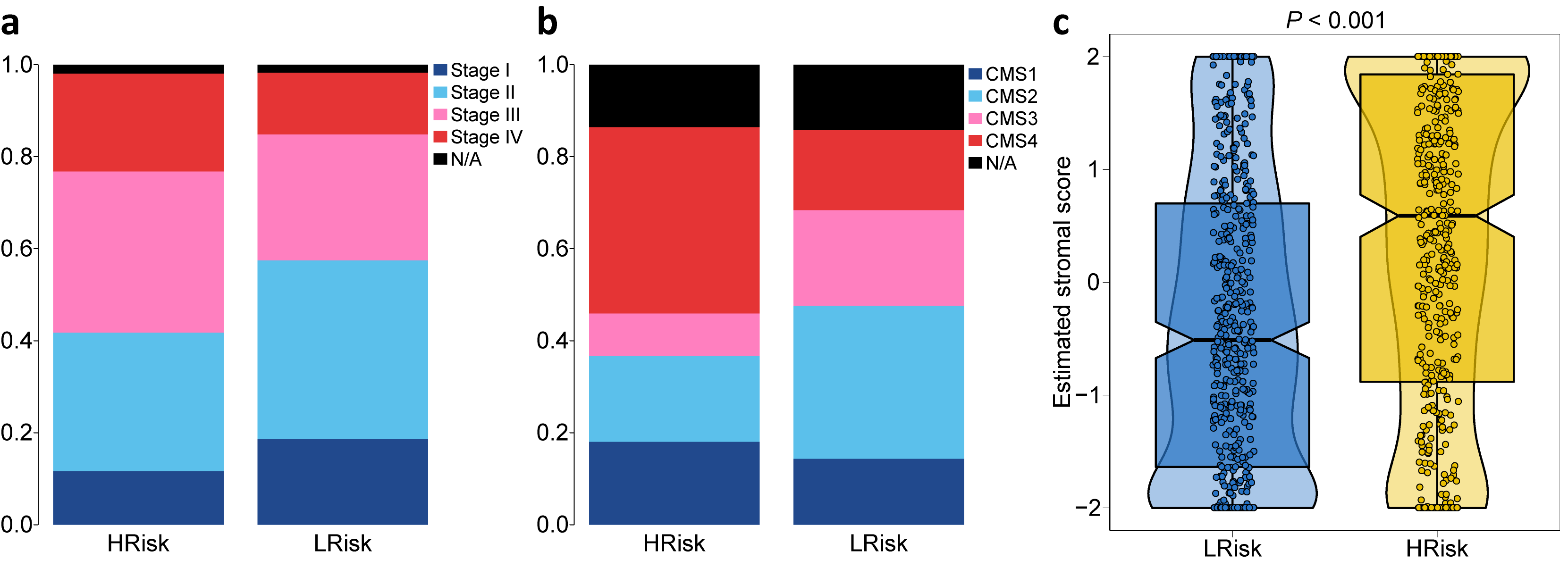

Supplement: Supplementary file 5 — Fig S5 [file CPR-53-e12861-s005.tif]

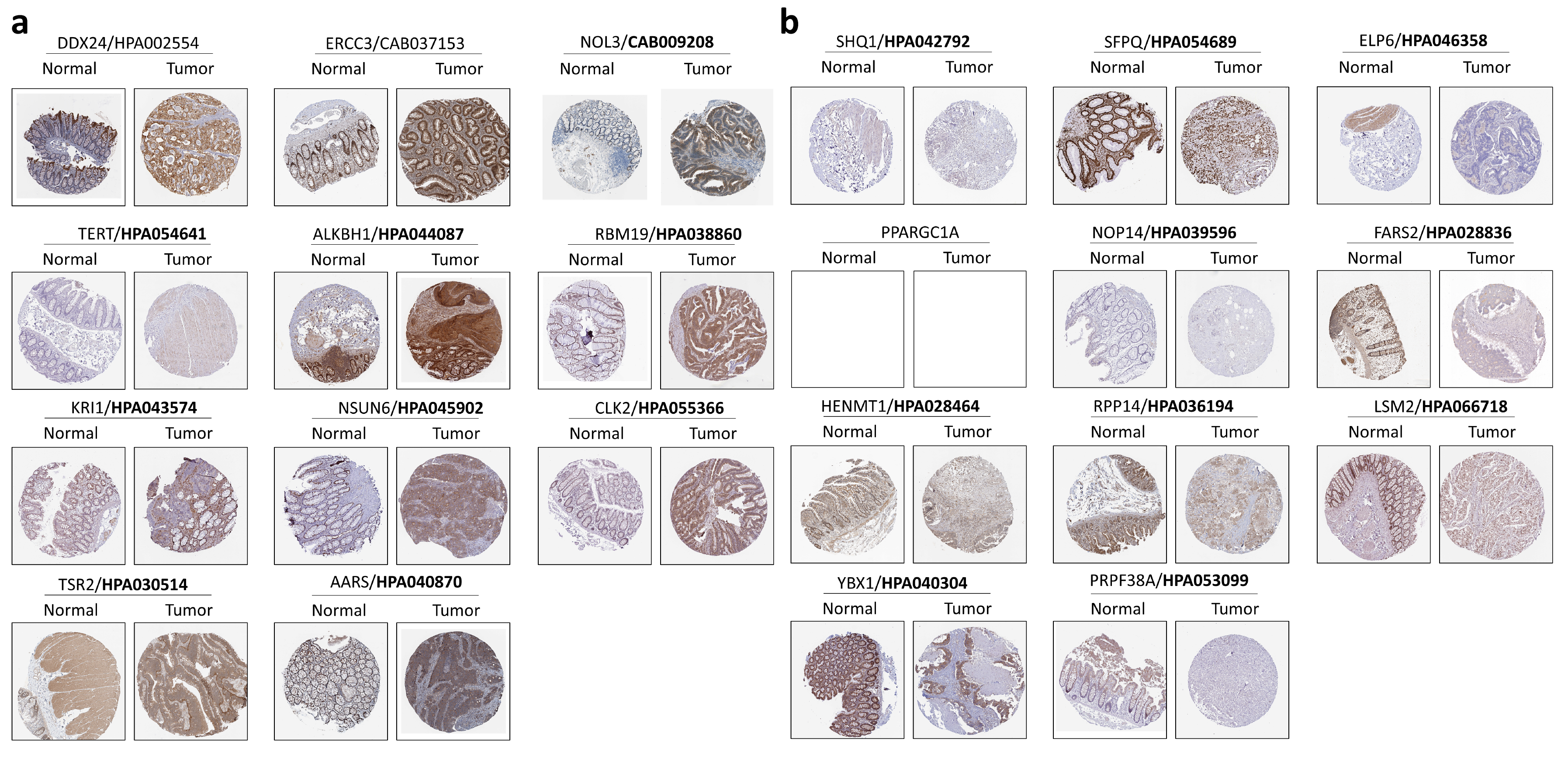

Supplement: Supplementary file 6 — Fig S6 [file CPR-53-e12861-s006.tif]

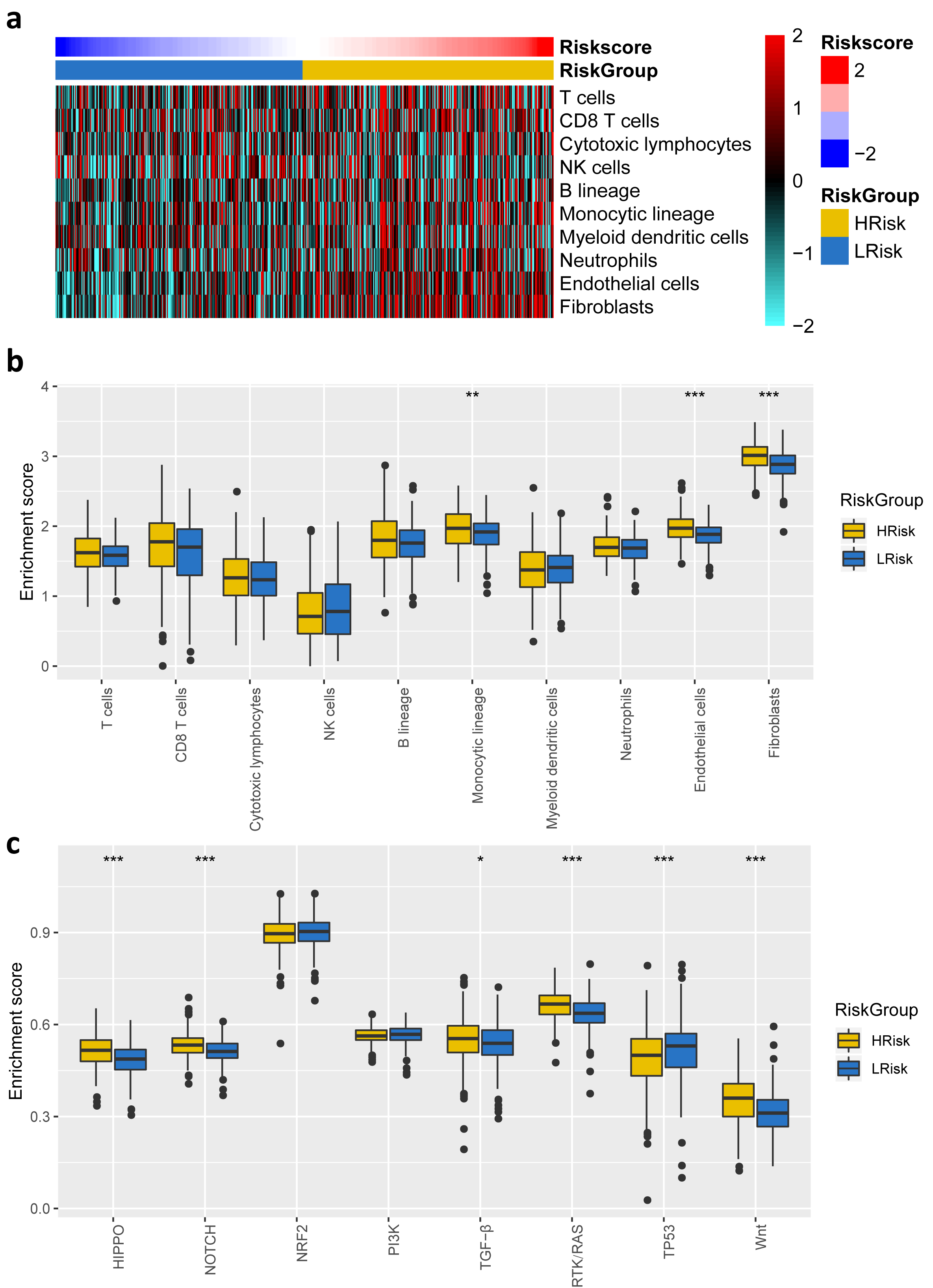

Supplement: Supplementary file 7 — Fig S7 [file CPR-53-e12861-s007.tif]

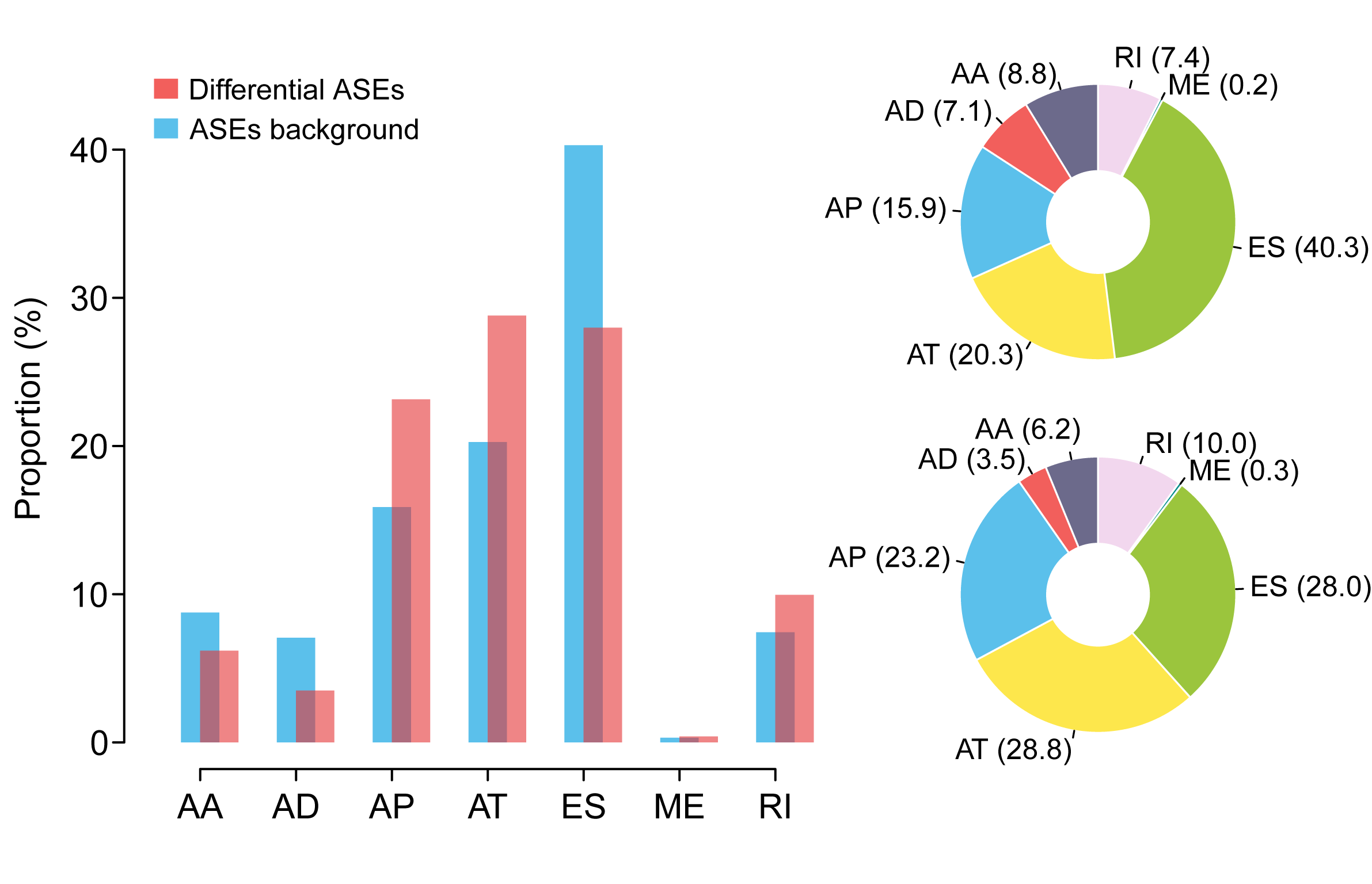

Supplement: Supplementary file 8 — Fig S8 [file CPR-53-e12861-s008.tif]
